# Assessment of Tachykinin Receptor 3′ Gene Polymorphism rs3733631 in Rosacea

**DOI:** 10.1155/2015/469402

**Published:** 2015-09-30

**Authors:** Anthony Karpouzis, Paschalis Avgeridis, Gregory Tripsianis, Elisavet Gatzidou, Niki Kourmouli, Stavroula Veletza

**Affiliations:** ^1^Department of Dermatology, Faculty of Medicine, Democritus University of Thrace, 68100 Alexandroupolis, Greece; ^2^Department of Medical Biology, Faculty of Medicine, Democritus University of Thrace, 68100 Alexandroupolis, Greece; ^3^Department of Medical Statistics, Faculty of Medicine, Democritus University of Thrace, 68100 Alexandroupolis, Greece

## Abstract

*Background.* Rosacea is a chronic skin disease, possibly following the neurogenic skin inflammation model. Neurokinin B, involved in the pathogenesis of Parkinson's disease, frequently coexisting with subsequent onset of rosacea, is an endogenous ligand of the tachykinin receptor 3 (TACR3). *Methods.* 128 rosacea patients and 121 matched controls were genotyped for rs3733631 by PCR-RFLP and analyzed by chi-square test. *Results.* We observed statistically significant predominance of the C/G or G/G genotype (*p* = 0.006) and of the G allele (*p* = 0.004) in the papulopustular (PP) form of rosacea and statistically marginal significance of the C/G or G/G genotype in erythematotelangiectatic (ET) rosacea (*p* = 0.052). Significantly higher frequency of the C/G or G/G genotype and G allele in PP rosacea (*p* = 0.021 and *p* = 0.008, resp.) was ascertained within male patients. *Conclusion.* TACR3 rs3733631 G allele possibly predisposes the evolution of the initial phase of rosacea to the PP and not the ET form in male patients.

## 1. Introduction

Rosacea is a persistent and recurrent inflammatory condition of the middle face as a rule. Classical morphology rosacea frequent subtypes are the erythematotelangiectatic (ET) rosacea and the papulopustular (PP) rosacea [1], while the other anatomoclinical forms are the granulomatous (or lupoid) rosacea, the phymatous rosacea or rhinophyma [2], and rosacea fulminans or pyoderma faciale [1]. In general, a persistent stasis erythema may precede by years the development and establishment of either an ET form or a PP form. Rosacea may appear in any immunocompetent patient and has to be differentiated from demodicosis of either immunocompetent or immunodeficient patients [2].

Semeiology, symptomatology, and pathologic features of rosacea suggest that its pathogenesis is mediated by sensory nerves via antidromically released neuromediators, therefore constituting a model of neurogenic inflammation [3].

After emotional stimulation or ingestion of alcohol, catecholamine is released from gastric mucosa or adrenal medulla and kallikrein is secreted by salivary glands, increasing bradykinin levels in blood. Bradykinin is associated with centrofacial vasodilatation [4]. Substance P (SP) is a neuropeptide, secreted by sensory neurons of human epidermis and dermal papillae. SP biologic functions consist in local axon reflex vasodilatation, acceleration of epidermal and endothelial cell proliferation, mast cell degranulation, antidromic vasodilatation, and liberation of inflammation mediators from macrophages and T lymphocytes. Biopsies on papular lesion and healthy skin of volunteer rosacea patients have shown that, in genetically predisposed persons, SP vasodilatory activity may be involved in the induction and/or maintenance of PP rosacea [5].

Vessel dilatation in rosacea is not due to yielding to damaged connective tissue but to fusion of vessels by a gradual breakdown of adjacent vascular walls [6]. Vascular endothelial growth factor (VEGF) staining on skin sections has detected no differences between PP rosacea and ET rosacea [7]. Laser Doppler Imager has detected elevated blood flow in affected skin compared with nonaffected skin of the same patient in the PP form but not in the ET form of rosacea [8].

Normal skin* Demodex folliculorum* (a head sebaceous follicle lumen mite), usually located at the upper part of the infundibulum of the follicle [9], has been indirectly involved in rosacea, although a possible causal correlation has not been established up to this day. The majority of rosacea medications do not affect* Demodex* but do improve rosacea, while treatment with Lindane, which is effective on the mite, does not improve the condition [1]. Possibly,* Demodex* may be an exclusively exacerbating factor in rosacea patients. Density of* Demodex* was demonstrated to be significantly higher in the PP form of rosacea, although no correlation was ascertained between* Demodex* intrafollicular inflammation and perifollicular inflammation. Possibly increased blood flow in rosacea skin dilated vessels constitutes an ideal condition for dermis* Demodex* multiplication.* Demodex folliculorum* antigens reacting with rosacea patients' sera have been considered to be responsible for stimulating mononuclear cell proliferation [10]. High vascular density was significantly more frequent in the PP form skin sections. The number of mast cells (which promote vasodilatation, angiogenesis, and fibrosis) was significantly higher in lesional than in nonlesional skin of patients with rosacea [10].

All things considered, it is evident that neuropeptides such as SP and/or bradykinin may play a decisive role in increasing blood flow and/or vascular density in rosacea. Consequently, it looked reasonable to explore the involvement of single nucleotide polymorphisms (SNPs) within neuromediators and their receptors in rosacea.

“Kinin” is considered as hypotensive polypeptide, contracting most isolated smooth muscles. Tachykinins (TACs) are characterized by pharmacological similarity to their endogenous inflammatory mediator, bradykinin, yet TACs are not of similar structure to that of bradykinin. TACs are not kinins but the name remains [11]. Heparin-initiated bradykinin formation plays an important role in mast cell-mediated diseases [12], such as rosacea.

TACs are a family of closely related peptides such as SP, neurokinin A, and neurokinin B, which act as neuropeptides in nonneuronal cells and noninnervated tissues [13]. Biological actions of TAC1 and TAC3 are mediated by three types of G protein-coupled receptors (TACR1, TACR2, and TACR3). TACR3 or NK-R3 or neurokinin B receptor belongs to a family of genes which function as receptors for TACs, characterized by interaction with G proteins, variations in the 5-end of the sequence, and 7-hydrophobic transmembrane regions interactions. TAC1 gene is localized on chromosome 7q21-q22, TAC3 is localized on 12q13-q21, TACR1 is localized on 2p12, TACR2 is localized on 10q11-q21, and TACR3 is localized on 4q25 [14]. Circulating neurokinin B is elevated in women with preeclampsia during the third trimester of pregnancy [15]. The increased placental expression of TAC3 gene belongs to the mechanism responsible for the elevated circulating neurokinin B levels in preeclampsia [16]. In preeclampsia complicated pregnancies, elevated neurokinin B plasma levels have been associated with elevated nitric oxide metabolite levels possibly in a compensatory mechanism to improve blood flow to the uteroplacental unit [15]. In familial hypogonadotropic hypogonadism, mutations of neurokinin B gene and its respective receptor TACR3 suggested an eventual key role for neurokinin B in the central control of reproduction [17].

Rosacea is significantly more frequent in patients suffering from preexisting Parkinson's disease [18]. Neurokinin B (endogenous ligand of TACR3) is involved in pathogenesis of Parkinson's disease by interacting with brain dopaminergic transmission [19].

Kisspeptin/neurokinin B/dynorphin-expressing neurons of hypothalamus, promoting skin vasodilatation, participate in the estrogen modulation of the body temperature and contribute to the induction of ensuing hot flushes in menopausal women; such flushes are clinically similar to those experienced in rosacea [20]. The role of mast cells in tachykinin neurogenic skin inflammation induction has been shown and thus their intense presence in PP rosacea [21, 22] may be explained.

We chose to study the TACR3 gene polymorphism rs3733631, because TACR3 endogenous ligand, neurokinin B, by increasing blood flow (anatomoclinical feature of rosacea), is involved in the pathophysiology of (a) Parkinsonism (frequently coexists with rosacea) and (b) hot flushes in menopausal women (clinically similar to rosacea vasomotor crises).

Possible involvement of the TACR3 gene in rosacea would most probably be associated with either upregulation or downregulation of this gene. SNPs such as rs3733631, localized within the promoter area, upstream from the coding region, make prime candidates to reflect any association between gene and disease. Certain additional TACR3 polymorphisms, such as A29V, G59E, S455G, A449S, W275X, and rs4580655, have been associated with idiopathic central pubertal disorders [23, 24], predicting and improving learning and memory in aged organism [25], alcohol and cocaine dependence [26], and pediatric slow transit constipation [27].

## 2. Materials and Methods

### 2.1. Patients' Characteristics

One hundred and twenty-eight patients, examined at the Outpatient Clinic of the University Department of Dermatology at the University Hospital of Alexandroupolis, 53 males (41.4%) and 75 females (58.6%), were included in this study. Patients' age ranged from 14 to 86 years with a median age of 56.25 ± 16.35 years. All patients were clinically diagnosed by the same physician and suffered from either the ET form (*n* = 67) or the PP form (*n* = 61) of central face rosacea [1]. Rosacea patients with accompanying disease were excluded from the study to avoid possible interactions with potentially coexisting genetically determined disorders. One hundred and twenty-one unrelated subjects, free from rosacea, not suffering from any potentially genetically determined disorder, individually matched to patients by both gender and age, were recruited as controls [54 (44.6%) males and 67 (55.4%) females]. There were no significant differences in gender (*p* = 0.608) and age (*p* = 0.783) between patients and controls (Table 1). Work procedures conform with the Helsinki Declaration Principles.

### 2.2. Methods

Genomic DNA from whole blood was isolated by salting out as previously described [28]. We genotyped the TACR3 gene polymorphism rs3733631 C/G by PCR-RFLP as follows: primers F′: 5′-CTTGCAGCGAATGAATGAAA-3′ and R′: 5′-GGGTAATCGAGTCACATCAGG-3′ generated a 216p long amplicon after 40 cycles of PCR at 95° 30′′, 55° 30′′, and 72° 30′′. The PCR product was subsequently digested with restriction enzyme HaeIII which generates three bands 107, 86, and 21 bp for allele G and two bands 128 and 86 bp for allele C. Restriction digests were analyzed by electrophoresis in 2.5% agarose gel.

Statistical analysis of the data was performed using the statistical package for the Social Sciences (SPSS) (INC, Chicago, IL, USA). The chi-square test was used to assess differences in genotype and allele frequencies between patients suffering from rosacea and matched controls. It was also used to compare the observed frequency of each genotype with that expected for a population in the Hardy-Weinberg equilibrium. Multivariate unconditional logistic regression analysis was used to estimate age-adjusted odd rations (OR) and 95% confidence intervals (CI) as the measure of association of the studied polymorphism with the appearance of rosacea. All tests were two-tailed and statistical significance was considered for values less than 0.05.

## 3. Results

TACR3 gene polymorphism rs3733631 C/G is located at the promoter of TACR3. The distribution of genotypes and alleles in patients and healthy controls is shown in Table 2. C/C, C/G, and G/G genotypes were found in 84.4%, 13.3%, and 2.3% of patients and in 87.6%, 11.6%, and 0.8% of healthy controls, respectively. The genotype distribution was in Hardy-Weinberg equilibrium in controls (*χ* ^2^ = 0.481, df = 1, and *p* = 0.488) but not in patients (*χ* ^2^ = 4.512, df = 1, and *p* = 0.034). No statistically significant differences were detected in either genotype or allele frequencies between the two groups (*p* = 0.433 and *p* = 0.295, resp.). However, a trend of higher frequency of G carriers (G/G or C/G genotype) and G allele was substantiated within males (22.7% versus 13.0%, *p* = 0.139 for G/G, G/C genotypes, and 14.2% versus 6.5%, *p* = 0.071 for the G allele) (Table 2). More specifically, in male patients, an increased (but not statistically significant) risk of rosacea incidence was associated with G allele (OR, 2.38; 95% CI, 0.93–6.11). Further stratification for the clinical form of rosacea in each patient suggested a statistically significant predominance of G carriers suffering from PP form of rosacea versus controls (70.5% C/C, 26.2% C/G, and 3.3% G/G in patients versus 87.7% C/C, 11.6% C/G, and 0.8% G/G in controls) as well as a higher frequency of the allele G (83.6% C and 16.4% G in patients versus 93.4% C and 6.6% G in controls) (Tables 3(a) and 3(b)). Therefore, the frequency of C/G or G/G genotype on one hand is statistically significantly higher in PP rosacea patients (*p* = 0.006) and G allele is also more frequent in PP patients (*p* = 0.004, Table 3(b)). Consequently, a statistically significant increased risk of PP rosacea incidence was associated with G allele (OR, 2.77; 95% CI, 1.38–5.57). In patients with ET rosacea, the predominance of G allele was not significant (*p* = 0.087) but the higher frequency of C/G or G/G genotype in patients was closer to being statistically significant (*p* = 0.052) (Table 3(a)). Regarding the distribution of genotypes in PP rosacea males, the higher frequency of C/G or G/G genotype in patients was statistically significant (*p* = 0.021) and the frequency of G allele was significantly higher (*p* = 0.008, Tables 3(a) and 3(b)). No statistical significance was observed within females.

## 4. Discussion

Genetic polymorphisms have not been previously studied extensively in rosacea. Specifically, in one study of Bsm1 polymorphism of the vitamin D receptor (VDR), the frequency of allele 1 was increased in patients with rosacea fulminans, but that increase was not statistically significant. No differences were detected in PP or ET rosacea [29]. Another study showed a statistically significant relationship between both null combination of glutathione-S-transferase M1 (GCTM1) and GSTT1 genotype polymorphisms and rosacea, in the setting of a comparative genetic study, in a group of rosacea patients and a group of matched healthy controls [30].

A recently published genome-wide association study reported correlation of rosacea with HLA-DRB1^*∗*^, HLA-DQB1^*∗*^, and HLA-DQA1^*∗*^ as well as rs763035, providing evidence regarding contribution of genetic component to pathogenesis of rosacea [31].

In our study we observed a trend (*p* = 0.071) within male patients to carry G allele of rs3733631 with higher frequency than controls regardless of the type of rosacea. No such trend was observed within females.

There have been no previous studies in the literature reporting association or not of rs3733631 TACR3 gene polymorphism with rosacea; however, it has been studied in alcohol and cocaine dependence [26].

The Toll-like receptor 2 gene (TLR2) is located at 4q32 [32], adjacent to TACR3 at 4q25. In PP rosacea, serine protease kallikrein-5 (KLK5) and two abnormal cathelicidin peptides (LL-37 and FA-29) are upregulated in the skin and induce erythema and vascular dilatation when injected in mouse skin. LL-37 is characterized by lower antimicrobial activity than normal smaller cathelicidin in healthy skin and it promotes angiogenesis and consequently secondary inflammation [33].

It is possible that the polymorphism at the adjacent TACR3 may be somehow involved in increasing expression of TLR2 in rosacea and therefore in upregulation of KLK5 in a calcium-dependent manner; possibly PP rosacea patients overreact, resulting in the histogenesis of rosacea papules and pustules, even though bacterial diversity and quantities are similar in rosacea lesions and in normal skin [34].

TLR2 stimulation occurs only as a response to specific triggers, such as emotional stimulation or ingestion of alcohol and kallikrein, secreted by salivary glands, increasing vasodilatory bradykinin blood levels [5, 33]. The vitamin D-dependent amplification mechanism might induce increase of TLR2 susceptibility, as 1,25 (OH)_2_ vitamin D gene polymorphism has been correlated with the intensity of inflammation [29].

Polymorphism R702W in NOD2/CARD15 is specifically associated with childhood granulomatous rosacea [35]. This gene participates in the N-terminal caspase recruitment (NACT) protein domain family, being involved in the response of Toll-like receptors transduced inflammatory stimuli [36].

Our study suggested a statistically significant predominance of C/G or G/G genotype (and G allele) in patients suffering from PP rosacea versus matched healthy controls. The number of patients included in our study constitutes a relatively small size sample; additional studies are needed to confirm our reported results. PP rosacea is characterized by elevated blood flow and increased SP vasodilatory activity in affected skin. Genotypes carrying G allele may be accompanied by alterations of kisspeptin/neurokinin B/dynorphin (KNDy) neurons of the hypothalamus and these altered neurons (via projections to rostral hypothalamic structures such as the medial preoptic area and medial preoptic nucleus which control thermoregulatory effectors) could maximize the vasodilatation of the skin as well as the inflammation (induced by vasodilatation), resulting in development of PP rosacea [6, 20, 33].

## 5. Conclusion

Genotypes carrying G allele may provide a genetic predisposition to PP rosacea and not to the ET form. Therefore, PP rosacea may be characterized by a genetic uniqueness, posing the possibility of its nosological distraction from the ET form.

## Figures and Tables

**Table 1:** Characteristics of patients with rosacea and healthy controls.

	Patients	Controls	*p* value
Number	128	121	
Age (years; mean ± SD)	56.25 ± 16.35	56.81 ± 15.96	0.783
Gender			0.608
Male	53 (41.4%)	54 (44.6%)	
Female	75 (58.6%)	67 (55.4%)	
Type of disease			
PP	61 (47.7%)	—	
ET	67 (52.3%)	—	

**Table 2:** Distribution of genotypes among patients with rosacea and healthy controls.

	Controls^*∗*^	Patients^*∗*^	OR^†^	95% CI	*p* value
Total					
Genotype					
C/C	106 (87.7)	108 (84.4)	Ref.		
C/G	14 (11.6)	17 (13.3)	1.21	0.57–2.58	0.621
G/G	1 (0.8)	3 (2.3)	3.16	0.32–31.07	0.325
Recessive model					
C/C	106 (87.7)	108 (84.4)	Ref.		
C/G or G/G	15 (12.4)	20 (15.6)	1.34	0.65–2.76	0.433
Allele contrast					
C	226 (93.4)	233 (91.0)	Ref.		
G	16 (6.6)	23 (9.0)	1.43	0.73–2.78	0.295

Males					
Genotype					
C/C	47 (87.0)	41 (77.3)	Ref.		
C/G	7 (13.0)	9 (17.0)	1.48	0.50–4.34	0.478
G/G	0 (0.0)	3 (5.7)	—	—	—
Recessive model					
C/C	47 (87.0)	41 (77.3)	Ref.		
C/G or G/G	7 (13.0)	12 (22.7)	1.97	0.71–1.02	0.193
Allele contrast					
C	101 (93.5)	91 (85.8)	Ref.		
G	7 (6.5)	15 (14.2)	2.38	0.93–6.11	0.071

Females					
Genotype					
C/C	59 (88.1)	67 (89.3)	Ref.		
C/G	7 (10.4)	8 (10.7)	1.02	0.35–2.99	0.973
G/G	1 (1.5)	0 (0.0)	—	—	—
Recessive model					
C/C	59 (88.1)	67 (89.3)	Ref.		
C/G or G/G	8 (11.9)	8 (10.7)	0.9	0.32–2.54	0.835
Allele contrast					
C	125 (93.3)	142 (94.7)	Ref.		
G	9 (6.7)	8 (5.3)	0.8	0.30–2.13	0.65

Note: statistical significance for differences in genotype, G-containing genotype, and allelic frequencies between patients with rosacea and healthy controls: (i) *χ* ^2^  = 1.113, df = 2, and *p* = 0.573; *χ* ^2^ = 0.537, df = 1, and *p* = 0.464; *χ* ^2^ = 0.970, df = 1, and *p* = 0.325 among the entire cohort; (ii) *χ* ^2^ = 3.650, df = 1, and *p* = 0.161; *χ* ^2^ = 1.716, df = 1, and *p* = 0.190; *χ* ^2^ = 3.412, df = 1, and *p* = 0.065 among males; (iii) *χ* ^2^ = 1.127, df = 1, and *p* = 0.569; *χ* ^2^ = 0.057, df = 1, and *p* = 0.811; *χ* ^2^ = 0.241, df = 1, and *p* = 0.624 among females. ^*∗*^Data are number of subjects and percentage (%); ^†^adjusted for age and gender.

**(a):** 

	Controls^*∗*^	ET^*∗*^	OR^†^	95% CI	*p* value
Total					
Genotype					
C/C	106 (87.7)	65 (97.0)	Ref.		
C/G	14 (11.6)	1 (1.5)	0.12	0.02–0.93	0.043
G/G	1 (0.8)	1 (1.5)	1.72	0.11–28.25	0.704
Recessive model					
C/C	106 (87.7)	65 (97.0)	Ref.		
C/G or G/G	15 (12.4)	2 (3.0)	0.22	0.05–1.01	0.052
Allele contrast					
C	226 (93.4)	131 (97.8)	Ref.		
G	16 (6.6)	3 (2.2)	0.33	0.10–1.17	0.087

Males					
Genotype					
C/C	47 (87.0)	23 (92.0)	Ref.		
C/G	7 (13.0)	1 (4.0)	0.31	0.04–2.68	0.285
G/G	0 (0.0)	1 (4.0)	—	—	—
Recessive model					
C/C	47 (87.0)	23 (92.0)	Ref.		
C/G or G/G	7 (13.0)	2 (8.0)	0.62	0.12–3.26	0.57
Allele contrast					
C	101 (93.5)	47 (94.0)	Ref.		
G	7 (6.5)	3 (6.0)	0.97	0.24–3.95	0.966

Females					
Genotype					
C/C	59 (88.1)	42 (100.0)	—		
C/G	7 (10.4)	0 (0.0)	—	—	—
G/G	1 (1.5)	0 (0.0)	—	—	—
Recessive model					
C/C	59 (88.1)	42 (100.0)	—		
C/G or G/G	8 (11.9)	0 (0.0)	—	—	—
Allele contrast					
C	125 (93.3)	84 (100.0)	—		
G	9 (6.7)	0 (0.0)	—	—	—

Note: statistical significance for differences in genotype, G-containing genotype, and allelic frequencies between patients with ET rosacea and healthy controls: (i) *χ* ^2^ = 6.089, df = 2, and *p* = 0.048; *χ* ^2^ = 4.644, df = 1, and *p* = 0.031; *χ* ^2^ = 3.437, df = 1, and *p* = 0.064 among the entire cohort; (ii) *χ* ^2^ = 3.563, df = 2, and *p* = 0.168; *χ* ^2^ = 0.417, df = 1, and *p* = 0.518; *χ* ^2^ = 0.013, df = 1, and *p* = 0.908 among males; (iii) *χ* ^2^ = 5.412, df = 2, and *p* = 0.067; *χ* ^2^ = 5.412, df = 1, and *p* = 0.020; *χ* ^2^ = 5.885, df = 1, and *p* = 0.014 among females. ^*∗*^Data are number of subjects and percentage (%); ^†^adjusted for age and gender.

**(b):** 

	Controls^*∗*^	PP^*∗*^	OR^†^	95% CI	*p* value
Total					
Genotype					
C/C	106 (87.7)	43 (70.5)	Ref.		
C/G	14 (11.6)	16 (26.2)	2.81	1.26–6.27	0.011
G/G	1 (0.8)	2 (3.3)	5	0.44–56.98	0.195
Recessive model					
C/C	106 (87.7)	43 (70.5)	Ref.		
C/G or G/G	15 (12.4)	18 (29.5)	2.96	1.37–6.41	0.006
Allele contrast					
C	226 (93.4)	102 (83.6)	Ref.		
G	16 (6.6)	20 (16.4)	2.77	1.38–5.57	0.004

Males					
Genotype					
C/C	47 (87.0)	18 (64.3)	Ref.		
C/G	7 (13.0)	8 (28.6)	2.94	0.93–9.32	0.067
G/G	0 (0.0)	2 (7.1)	—	—	—
Recessive model					
C/C	47 (87.0)	18 (64.3)	Ref.		
C/G or G/G	7 (13.0)	10 (35.7)	3.7	1.22–11.22	0.021
Allele contrast					
C	101 (93.5)	44 (78.6)	Ref.		
G	7 (6.5)	12 (21.4)	3.9	1.44–10.59	0.008

Females					
Genotype					
C/C	59 (88.1)	25 (75.8)	Ref.		
C/G	7 (10.4)	8 (24.2)	2.7	0.88–8.24	0.082
G/G	1 (1.5)	0 (0.0)	—	—	—
Recessive model					
C/C	59 (88.1)	25 (75.8)	Ref.		
C/G or G/G	8 (11.9)	8 (24.2)	2.36	0.80–7.01	0.121
Allele contrast					
C	125 (93.3)	58 (87.9)	Ref.		
G	9 (6.7)	8 (12.1)	1.92	0.70–5.22	0.204

Note: statistical significance for differences in genotype, G-containing genotype, and allelic frequencies between patients with PP rosacea and healthy controls: (i) *χ* ^2^ = 8.217, df = 2, and *p* = 0.016; *χ* ^2^ = 7.999, df = 1, and *p* = 0.005; *χ* ^2^ = 8.709, df = 1, and *p* = 0.003 among the entire cohort; (ii) *χ* ^2^ = 7.517, df = 2, and *p* = 0.023; *χ* ^2^ = 5.808, df = 1, and *p* = 0.016; *χ* ^2^ = 8.044, df = 1, and *p* = 0.005 among males; (iii) *χ* ^2^ = 3.696, df = 2, and *p* = 0.158; *χ* ^2^ = 2.490, df = 1, and *p* = 0.115; *χ* ^2^ = 1.661, df = 1, and *p* = 0.197 among females. ^*∗*^Data are number of subjects and percentage (%); ^†^adjusted for age and gender.
